# Supplementary figures and images for: Antibacterial Activity of an FtsZ Inhibitor Celastrol and Its Synergistic Effect with Vancomycin against Enterococci In Vitro and In Vivo
Source: Microbiol Spectr. 2023 Jan 9;11(1):e03699-22. doi: 10.1128/spectrum.03699-22 (PMC9927571; doi:10.1128/spectrum.03699-22)

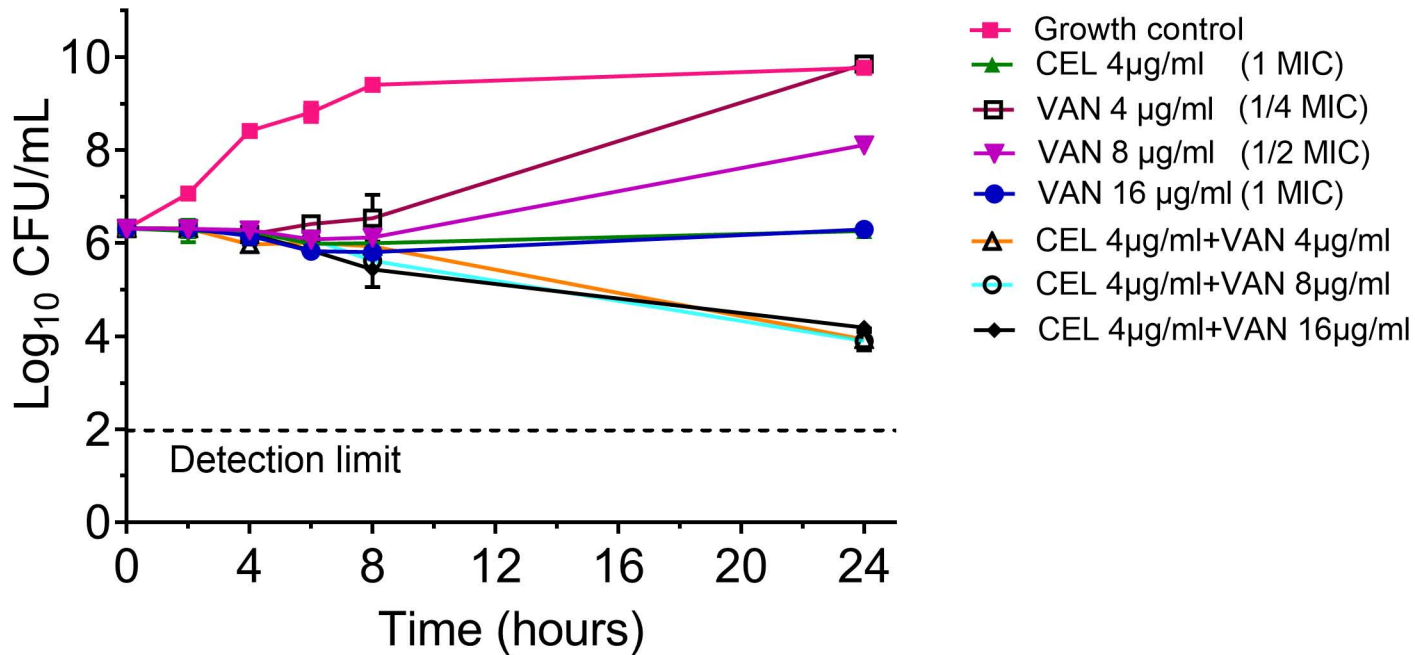

Supplement: Supplemental file 1 — Fig. S1. Download spectrum.03699-22-s0001.pdf, PDF file, 0.1 MB [file spectrum.03699-22-s0001.pdf]
